# The interplay between chemo-phoretic interactions and crowding in active colloids†

**DOI:** 10.1039/d2sm00957a

**Published:** 2023-02-10

**Authors:** Federico Fadda, Daniel A. Matoz-Fernandez, René van Roij, Sara Jabbari-Farouji

**Affiliations:** a Institute of Physics, University of Amsterdam 1098 XH Amsterdam The Netherlands fede.fadda1110@gmail.com s.jabbarifarouji@uva.nl; b Institute of Theoretical Physics, Faculty of Physics, University of Warsaw, Pasteura 5 02-093 Warsaw Poland daniel.matoz@fuw.edu.pl; c Institute for Theoretical Physics, Center for Extreme Matter and Emergent Phenomena, Utrecht University, Princetonplein 5 Utrecht 3584 CC The Netherlands r.vanroij@uu.nl

## Abstract

Many motile microorganisms communicate with each other and their environments *via* chemical signaling which leads to long-range interactions mediated by self-generated chemical gradients. However, consequences of the interplay between crowding and chemotactic interactions on their collective behavior remain poorly understood. In this work, we use Brownian dynamics simulations to investigate the effect of packing fraction on the formation of non-equilibrium structures in a monolayer of diffusiophoretic self-propelled colloids as a model for chemically active particles. Focusing on the case when a chemical field induces attractive positional and repulsive orientational interactions, we explore dynamical steady-states of active colloids of varying packing fractions and degrees of motility. In addition to collapsed, active gas, and dynamical clustering steady-states reported earlier for low packing fractions, a new phase-separated state emerges. The phase separation results from a competition between long-range diffusiophoretic interactions and motility and is observed at moderate activities and a wide range of packing fractions. Our analysis suggests that the fraction of particles in the largest cluster is a suitable order parameter for capturing the transition from an active gas and dynamical clustering states to a phase-separated state.

## Abstract 2

Competition between long-range attractive chemophoretic interactions and self-propulsion in active colloids leads to a new kind of phase separation at moderate activities and low densities that we call chemo-motility induced phase separation.

## Graphical abstract

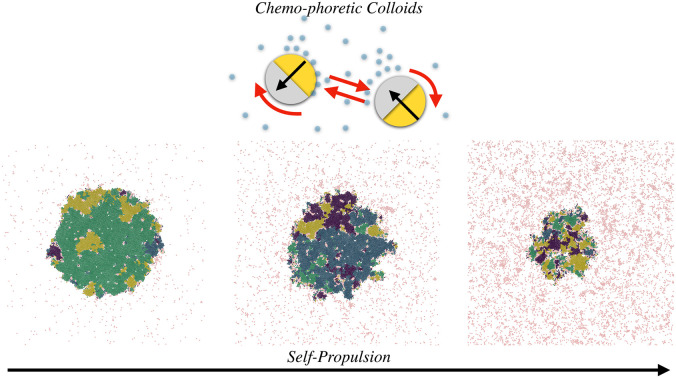

## I. Introduction

Active materials consisting of self-driven units exhibit a spectacular wealth of collective phenomena.^1–3^ A few examples include the emergence of flocking transition in birds, swarming in bacteria,^4–6^ and dynamical clusters in active colloids.^7^ The interplay between self-propulsion and many-body interactions in active units drives the formation of non-equilibrium structures which have no counterparts in equilibrium systems. For example, self-propulsion in conjunction with purely short-range repulsive interactions leads to motility-induced phase separation where large clusters of active particles coexist with a gaseous background;^8–17^ it is a true hallmark of the non-equilibrium nature of active matter.

Active units interact not only *via* direct forces resulting *e.g.* from steric or contact interactions but also *via* field-mediated interactions.^18–28^ Examples include mediation *via* the surrounding medium,^18–22^ and chemo- or thermo-phoretic fields,^23–27^ which often lead to long-range and anisotropic interactions. The effects of short-range interactions such as excluded volume and attractive interactions on non-equilibrium structure formation have been a subject of intensive research,^22,29–38^ and there is some consensus about their effects on the collective behavior even if there are open issues about the statistical nature of dynamical phase transitions and their universality classes.^31,32,35^ However, the significance of long-range hydrodynamic and phoretic interactions leading to asymmetrical interactions on phase separation and collective dynamics is still a matter of debate^19,21,23–26,38^ and remains elusive. In particular, the interplay between long-range phoretic interactions and crowding has received less attention.^23,24^ The particle-resolved studies of chemo-phoretically interacting active colloids have so far concentrated on dilute and small systems.^23,39–41^ The continuum approaches such as kinetic theories^40,42^ are valid at best for moderate densities. To our knowledge, the interplay between crowding effects and phoretic interactions has not been explored so far.

**Fig. 1:**
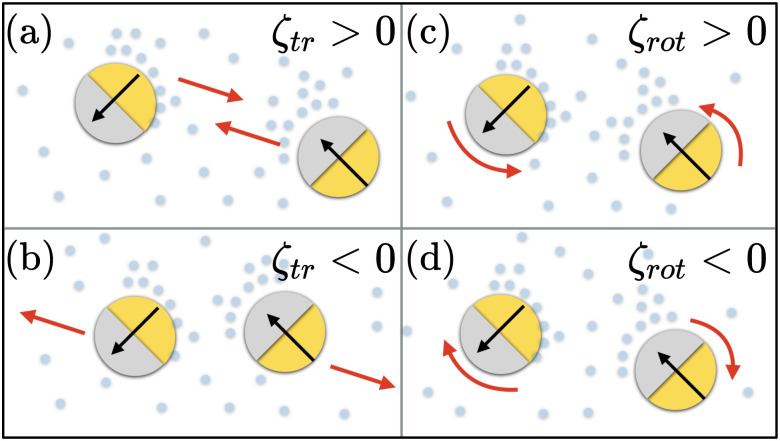
Schematics of chemical field mediated interactions between self-catalytic Janus colloids for different cases of translational *ζ*_tr_ and rotational *ζ*_rot_ chemotactic mobility parameters in the situation when the colloids act as chemical sinks. (a) When *ζ*_tr_ > 0, the colloids move towards each other, (b) when *ζ*_tr_ < 0, the colloids move away from each other, (c) when *ζ*_rot_ > 0 the colloids rotate towards each other, and (d) when *ζ*_rot_ < 0 they rotate away from each other.

In this work, we go beyond the state of the art and study the role of the density of active colloids interacting *via* steric interactions and chemical fields on non-equilibrium structure formation using particle-based computer simulations. We also investigate the nature of dynamical phase transitions from one steady-state to another as a function of packing fraction at different degrees of activity. The system that we focus on is a dispersion of heavy catalytic Janus colloids^7,43,44^ that settle to the bottom of the experimental cell to create a colloidal monolayer. The self-propulsion of catalytic Janus colloids is driven by self-diffusiophoresis,^45,46^ which creates a non-uniform chemical field around the particles and mediates long-range chemo-phoretic interactions.^47,48^ We adopt a minimal model of active Brownian particles where translational and orientational dynamics of particles are coupled to the chemical field,^23,39^ see Fig. 1. To overcome the challenge of simulating a large number of particles with long-range interactions, we have introduced the chemical-mediated interactions in an efficient Brownian dynamics GPU-implemented code,^49^ which allows for investigation of collective dynamics at high packing fractions.

**Fig. 2:**
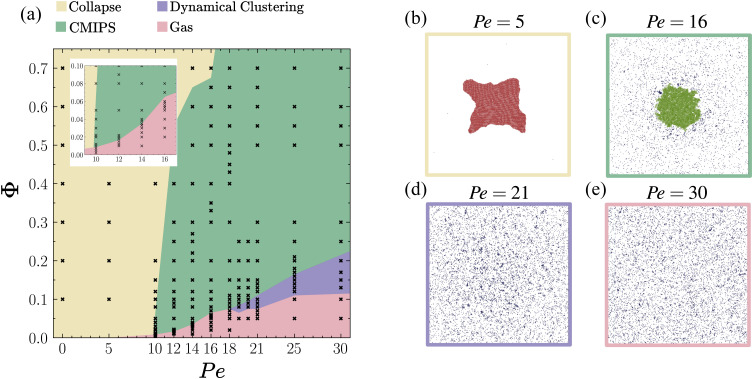
(a) State diagram of self-phoretic colloids in the Péclet-packing fraction (*Φ*–Pe) representation for translational and rotational mobility coefficients *ζ*_tr_ = 15.4 and *ζ*_rot_ = −0.38, respectively. We distinguish four distinct dynamical states: active gas (red region), dynamical clusters (green region), CMIPS (yellow region) and collapsed state (blue region). Panels (b–e) depict representative snapshots for collapsed, chemotactic phase separated, dynamical clustering and active gas states, respectively, obtained at fixed *Φ* = 0.1 but different propulsion speeds Pe = 5, 16, 21 and 30 as given on the top of each snapshot.

We have investigated the state-diagram of active chemo-phoretic colloids upon varying the Péclet number and packing fraction in the case when the chemical field induces attractive positional and repulsive orientational interactions as presented in Fig. 2. We find a new phase-separated state in which a dilute active gas coexists with a dense giant cluster occurring at a surprisingly low overall packing fraction *Φ* ∼ 10^−2^. Although the giant cluster structurally resembles the dense phase of the paradigmatic case of motility-induced phase separation (MIPS),^8–12^ here the underlying mechanisms of phase separation are different. It is induced by a competition between long-range attractive chemo-phoretic interactions driving the collapse and activity which leads to evaporation of particles from the surface of the giant collapsed cluster. Hence, we refer to this phase-separated state as chemo-motility induced phase separation (CMIPS).

The remainder of the article is organized as follows. In section II, we describe the physical model for chemotactic particles and numerical details of our particle-based simulations. In section III, we present our state diagram as a function of the reduced Péclet number and packing fraction and discuss the distinct signatures of each dynamical steady-state. In section IV, we investigate the characteristics of dynamical phase transitions by analyzing various order parameters including the fraction of particles in the largest cluster and the hexatic order parameter. We find that the mean fraction of particles in the largest cluster is a suitable order parameter characterizing the dynamical phase transitions. This parameter changes discontinuously, upon increasing the packing fraction, when going from the active gas to the CMIPS state at intermediate self-propulsion speeds, whereas it changes continuously at larger activities where the system undergoes a transition from a dynamical clustering state to the CMIPS state. Finally, we conclude our work in section V with a summary of our most important finding and directions for future work.

## II. Simulation details

### A. Dynamical equations

We consider a system of *N* spherical catalytic Janus colloids of radius *a* confined in a two-dimensional square box. The chemically-active parts of the Janus colloids initiate reactions which generate chemical products. Hence, the asymmetric distribution of the chemical products around a colloid leads to a phoretic slip velocity at the particle's interfacial layer, which self-propels the colloid. A Janus colloid, labelled with index *i*, self-propels at a speed *ν*_0_ along direction **e**_*i*_ fixed in the particle frame (Fig. 1). The self-propulsion speed is determined by the chemical activity and mobility of the two parts of the Janus colloid^45,50^ and in principle also depends on the chemical concentration. However, when chemical fuels are abundant and the release of the chemical products is rapid, one expects *ν*_0_ not to change noticeably due to local inhomogeneities of chemical field *c*(**r**) and, thus, we consider it as a fixed control parameter.^7,39,45,46^ In addition, the same chemical gradient field **∇***c*(**r**) that acts as a driving force for the propulsion of colloids induces translational and rotational drift velocities which mediate diffusiophoretic interactions between chemical-consuming colloids. Under these considerations, for the case of half-coated Janus colloids, the translational and rotational drift velocities can be written as^47^1
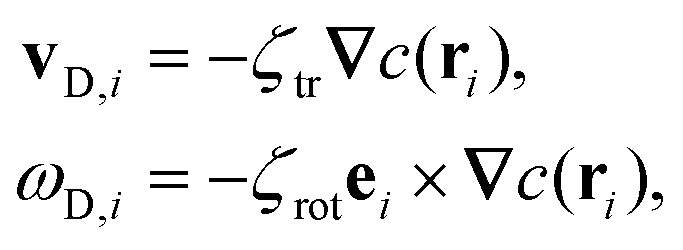
in which *ζ*_tr_ and *ζ*_rot_ are the translational and rotational phoretic mobility coefficients that encode the active colloids' response to the chemical field gradients. If *ζ*_tr_ > 0, colloids move away from the direction of local chemical gradient while for *ζ*_tr_ < 0 the particles move towards regions of higher concentration of chemicals. Likewise, for *ζ*_rot_ > 0, the colloids rotate away from the direction of the local gradient, whereas for *ζ*_rot_ < 0 they reorient in the direction of the chemical gradient (see Fig. 1).

In what follows, we neglect the hydrodynamic interactions and model the collective motion of phoretically interacting active colloids with positions **r**_*i*_ = *x*_*i*_**ê**_*x*_ + *y*_*i*_**ê**_*y*_ and orientations **e**_*i*_ = cos *φ*_*i*_**ê**_*x*_ + sin *φ*_*i*_**ê**_*y*_ in the overdamped limit, describing it using the following Brownian dynamics equations^23^2
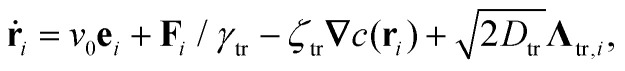
3
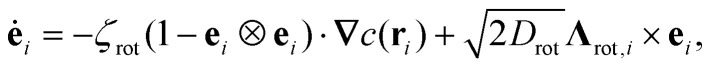
where *γ*_tr_ is the translational drag coefficient, and **Λ**_tr,*i*_ and **Λ**_rot,*i*_ are translational and rotational white noises with zero mean and unit variance, *i.e.*, 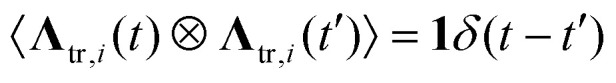 and 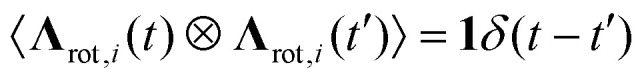. The term 
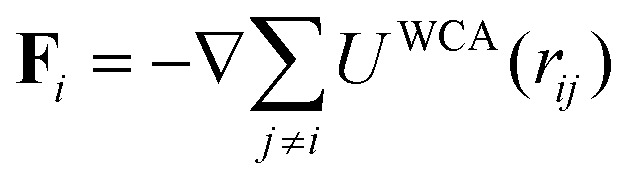
 corresponds to the force on the *i*-th particle due to excluded volume repulsive interactions from all the other particles which is modelled using the Weeks–Chandler–Anderson potential,^51^4
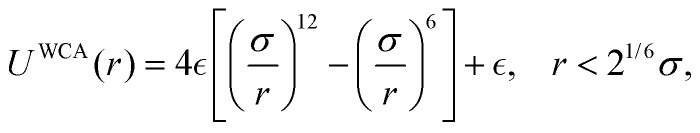
with *σ* = 2*a* the particle diameter, and 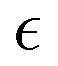 sets the strength of the potential. Note that, unlike ref. 23, 39 and 52 where the authors implemented a manual repulsion between particles separating them along the line connecting their centers in the case of overlap *r* < 2*a*, we introduce explicitly the WCA potential to account for the particle excluded volume. As can be seen from eqn (2) and (3), the self-propelled particles are coupled to the chemical field *c*(**r**,*t*) that represents the coarse-grained continuum concentration of the involved chemical species at time *t*. We assume that the chemical field diffuses in an infinite three-dimensional half-space with a diffusion coefficient *D*_c_ and has sinks at the positions of the particles since they consume the chemicals at rate *k* such that *c*(**r**,*t*) satisfies the following reaction diffusion equation5
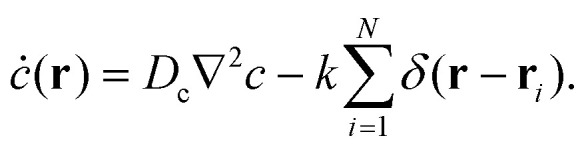
Typically, the chemical field diffuses much faster than the colloids such that *D*_c_ ≫ *D*_tr_. Hence, we can neglect the time dependence of the chemical field equation and adopt a stationary solution given using the Poisson equation and its solution in 3D,6
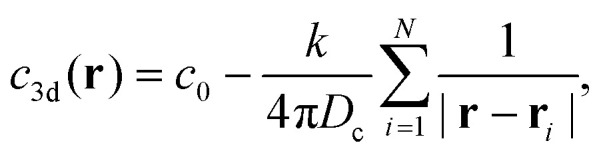
where *c*_0_ is the background chemical concentration. Our assumption of constant self-propulsion speed implies that *c*_0_ is sufficiently large so that *ν*_0_ ∝ *c*_0_.

Note that eqn (6) implies that each colloid instantly establishes a stationary long-range chemical sink around itself, which moves with it. The effective two-dimensional concentration field, in which the colloidal monolayer lives, can be approximately obtained by integrating over a thin layer of thickness *h* = 2*a* yielding *c*_2d_(**r**) ≈ *hc*_3d_(**r**).

Since the active colloids consume the chemicals, *ζ*_tr_ > 0 (*ζ*_tr_ < 0) leads to an effective attraction (repulsion) between colloids as the particles move towards (away from) the neighboring colloidal sinks while moving away from (toward) the concentrated regions of chemicals. Considering the form of stationary chemical field in eqn (6) the effective attractive (repulsive) interparticle potential is proportional to 
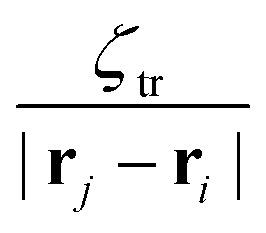
. Likewise, for *ζ*_rot_ > 0, particles rotate toward chemical sinks (other colloids), thus giving rise to alignment between colloids whereas *ζ*_rot_ < 0 leads to an interparticle misalignment. From eqn (3), we note that the interparticle orientational potential on colloid *i* is proportional to 
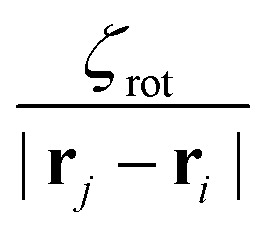
. The torque on particle *i* only depends on its own orientation **e**_*i*_ and its angle with interparticle vector **r**_*j*_ − **r**_*i*_ and not the orientation of the other colloid *j*. Therefore, chemotactic interactions lead to non-reciprocal orientational interactions.

### B. Dimensionless equations of motion

To carry out many-body simulations, we first render eqn (2), (3) and (6) dimensionless. We choose *t*_r_ = 1/(2*D*_rot_), 
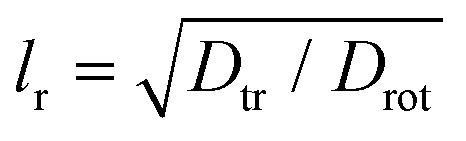
 and 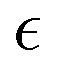 as units of time, length and energy, respectively. Thus, the dimensionless length and time units are defined as r* = **r**/*l*_r_ and t* = *t*/*t*_r_. The equations of motion in reduced units become7
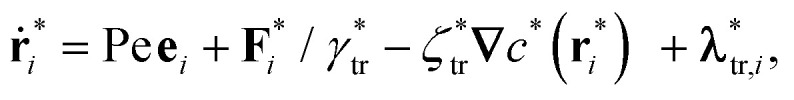
8
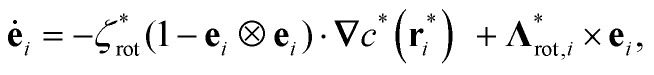
in which 
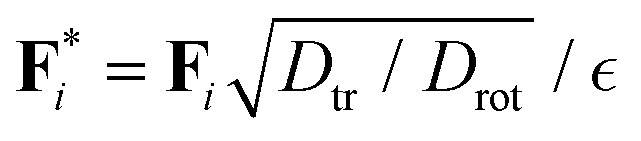
, 
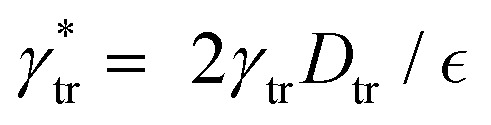
, 
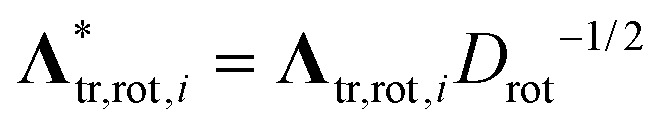
. The reduced two-dimensional concentration field is defined as 
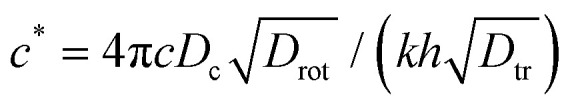
. The essential dimensionless parameters appearing in the reduced equations include the reduced self-propulsion speed called the Péclet number 
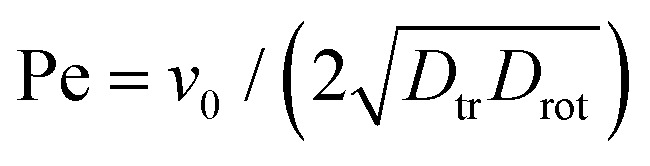
, and the reduced translational and rotational chemotactic coefficients 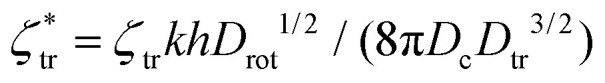 and 
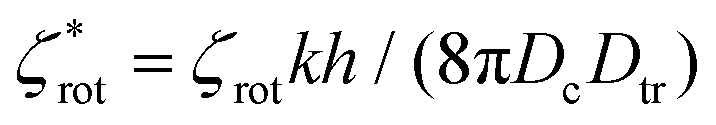
.

In summary, four dimensionless parameters determine the collective dynamics of self-phoretic colloids: the two chemotactic constants 
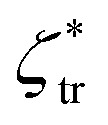
 and 
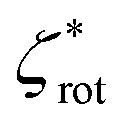
, the packing fraction *Φ* = *N*π*a*^2^/*L*^2^ with *L* being the linear size of the system and the Péclet number Pe. One checks that the reduced self-propulsion speed Pe can be rewritten as the ratio of the diffusive timescale to that of advection by active force, Pe = *τ*_diff_/*τ*_adv_. In our study we focus on varying the Péclet number and packing fraction while keeping all other parameters fixed. We note that, due to our choice of length and timescales, the definition of Pe used here differs by a constant prefactor from the typical studies of active colloids,^7,30,53^ where it is defined as 
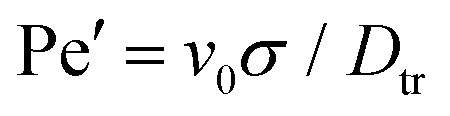
. Usually, it is assumed that the two diffusive coefficients follow the equilibrium relation *D*_rot_ = 3*D*_tr_*σ*^2^. Employing this relation, we find that 
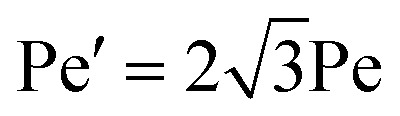
. Thus the two Péclet number definitions only differ by a numerical prefactor and can be easily mapped to each other for the sake of comparison with the literature using the latter definition.^7,30,53^

### C. Implementation of Brownian dynamics simulations

To integrate the many-body equations of motion eqn (7) and (8) we employed the Euler–Maruyama scheme.^54^ Therefore, the positions and angles evolved during a time step d*t* according to:9
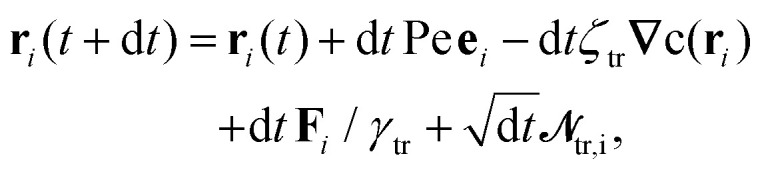
10
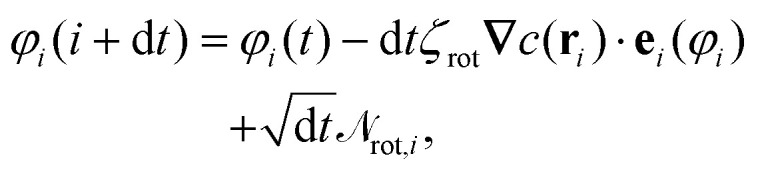
where for the ease of notation we have dropped the * superscripts from the dimensionless quantities and we will continue to do so in what follows. Here **𝒩**_tr,*i*_ = (𝒩^*x*^_tr,*i*_, 𝒩^*y*^_tr,*i*_). 𝒩^*x*^_tr,*i*_, 𝒩^*y*^_tr,*i*_ and 𝒩_rot,*i*_ denote the random variables representing the Gaussian white noise with unity variance. They are generated for each colloid at each time step using a normal distribution with a mean of zero and a standard deviation of one, respectively. To increase the efficiency of large-scale simulations, we added the chemical fields to a CUDA code for particle-based models.^49^ One demanding part of the simulations concerns the evaluation of the long-range chemical interactions between particles, where one needs to calculate the gradient of the chemical as the sum of *N* − 1 terms of the from 1/|**r**_*i*_ − **r**_*j*_| for each particle *i*. To solve for this, we have used a fast *N*-body algorithm that makes use of shared memory.^55^ In addition, to reduce further the computational costs, we updated the chemical field every 50 time-step. Within clusters of active colloids, the concentration field cannot freely diffuse. This leads to screening of the chemical field which is not taken into account by the equations of motion. To account for it, we introduce a manual screening rule whenever a colloid is surrounded by six closely packed neighbors similar to ref. 23, 39 and 52. Hence, whenever a colloid has six neighbors all located at a distance smaller than the screening length *ξ*, *i.e. r* ≤ *ξ*, we replace the term 1/*r* in eqn (6) with exp[−(*r* − *ξ*)/*ξ*], in which *r* = |**r**_*j*_ − **r**_*i*_| and *ξ* = 2*a*(1 + *δ*) with *δ* = 0.3.

### D. Simulation parameters

In our simulations, we set *σ* = 2, 
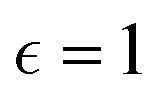
, *γ*_tr_ = 1 and the chemotactic mobility parameters to *ζ*_tr_ = 15.4 and *ζ*_rot_ = −0.38 while varying the reduced self-propulsion speed Pe and packing fraction *Φ* in the ranges 5 ≤ Pe ≤ 30 and 0.001 ≤ *Φ* ≤ 0.7. The chosen set of chemotactic parameters leads to effective interparticle attraction and orientational repulsion such that it leads to the formation of dynamical clusters for dilute systems^23^ similar to experiments of catalytic Janus colloids.^7,43^ We chose to set *N* = 10^4^ which allows for an efficient scan of the phase diagram in a reasonable time using a time-step in the range d*t* = 10^−5^−5 × 10^−5^. We observed that the steady-state is reached after *t* = (1 − 5) × 10^4^ in scaled units depending on the packing fraction. For small *Φ* and Pe the time to reach a steady-state is increased. As in ref. 23, 39, 52 and 56, we enclose the system with impenetrable walls. Therefore, when particles collide with the walls (*i.e.* |**r**_*i*_ − **r**_wall_| < *a*), they are reflected randomly away from them into the simulation box. Moreover, to compare the collective dynamics of chemo-phoretic active colloids with active Brownian particles (ABP), we also have carried out simulations of ABPs without phoretic interactions, *i.e.*, *ζ*_tr_ = *ζ*_rot_ = 0 with otherwise identical parameters for packing fractions 0.05 < *Φ* ≤ 0.7. For ABPs, we used periodic boundary conditions as in the absence of long-range attractive interactions, in dense ABPs, particles accumulate on the walls.^57^

For each state point, we ran simulations for 5 independent configurations for each set of fixed parameters. The simulations were continued until the system reached a steady-state which is defined by having small fluctuations of instantaneous mean cluster size and maximum cluster size. After reaching the steady state, the desired quantities were computed by time averaging over snapshots and over independent simulation runs.

### E. Analysis of steady-state configurations

#### Clustering algorithm

To determine the clusters in the system, we first identified the neighbours of each particle within a threshold cutoff *r*_c_ by using *k*-d trees.^58^ Then, we constructed a undirected graph that labels the connected particles by cluster-id and cluster-size.^59,60^ Using this information then is easy to construct several useful quantities. We first calculated the normalized distribution *P*(*n*) of clusters of any size *n* as 
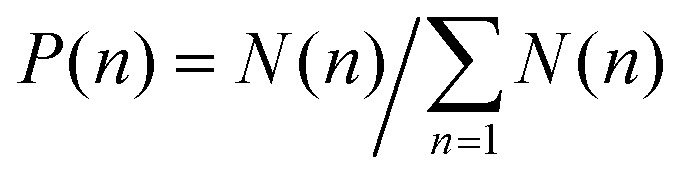
, where *N*(*n*) is the number of clusters of any size *n*. The mean cluster size for any snapshot is given as11
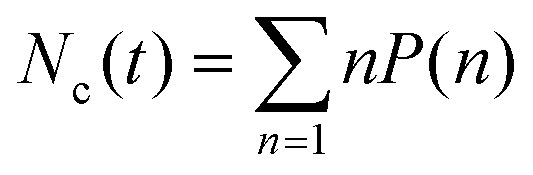
where 
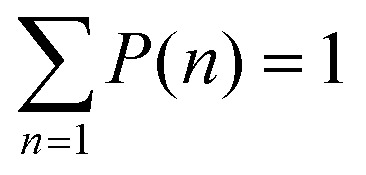
. In addition, we obtained the time-averaged cluster size *N*^avg^_c_, and the mean number of particles in the largest cluster *N*^max^_c_ by averaging over many steady-state snapshots. The mean fraction of particles in the largest cluster *N*^max^_c_/*N* can be used as an order parameter^28,61,62^ characterizing the transition from gas or dynamical clustering regimes to the phase-separated state.

#### Computation of local packing fraction distribution

Following ref. 10 and 63, we discretize the system by dividing it into squares of linear size *d*, so that continuous space is now replaced with discrete lattice containing (*L*/*d*)^2^ sites. Then, we constructed a discrete density, *φ̄*(**r**), defined for discrete positions **r** located at the center of each grid square given as12
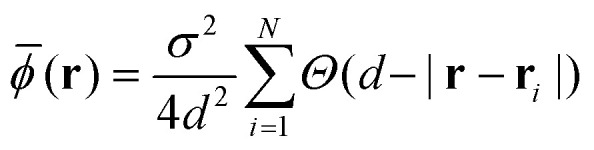
where *Θ*(*y*) is the Heaviside function. The size of the grid *d* was chosen to be 4*σ* to produce smooth probability distributions.

#### Hexatic order parameter

The global hexatic order parameter, also known as the six-fold bond orientational order parameter, is defined as^64^13
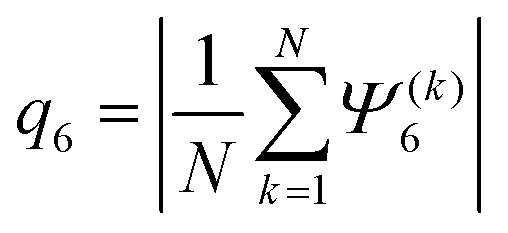
with14
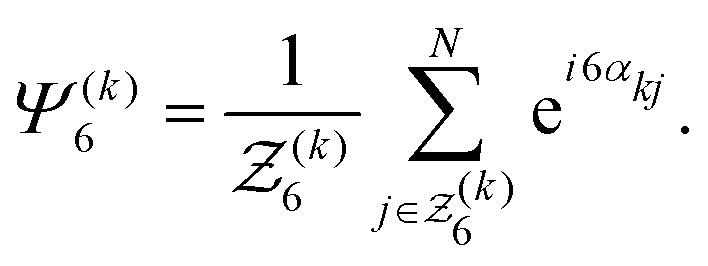
Here, 𝒵^(*k*)^_6_ is the number of nearest neighbours of the particle *k* and *α*_*kj*_ is the angle between the vector connecting the particle *k* to *j* and the horizontal *x*-axis. Given this definition, the parameter *q*^(*k*)^_6_ = 1 if a particle is surrounded by 6 closely packed neighbours in a system with perfect hexatic order. We also analyzed the distribution of the hexatic domains within the dense cluster of the CMIPS state by discretizing the argument of *Ψ*^(*k*)^_6_ in the range [0,2π] into bins *n* = 4.^33^ Furthermore, we discarded particles for which the hexatic order parameter was |*Ψ*^(*k*)^_6_| < 0.5 or belonged to hexatic domains containing less than 5% of the largest cluster.

## III. Dynamical steady-states of chemotactic colloids

### A. State diagram Pe − *Φ*

We start by giving an overview of our state diagram as a function of the dimensionless self-propulsion speed, *i.e.*, the Péclet number 
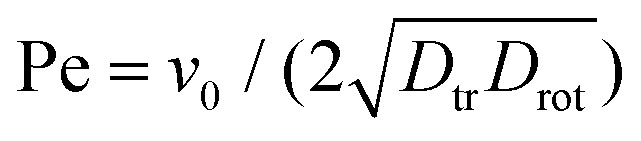
 and the packing fraction *Φ*, while fixing the translational and rotational phoretic mobility parameters. Previous studies of self-diffusiophoretic colloids have focused on dilute systems at moderate to high Pe while varying chemotactic parameters.^23,39^ It is found that the balance of translational phoretic attraction and rotational repulsion leads to the formation of pronounced dynamical clusters at low densities and moderate Péclet numbers.^23^ The long-range phoretic attractive forces ∝ *ζ*_tr_/*r*^2^ lead to the formation of clusters, while repulsive phoretic torque ∝ *ζ*_rot_/*r*^2^ rotates the swimming direction of active colloids that have joined a cluster outward, thus leading to their escape from clusters at sufficiently high Pe and formation of finite-sized dynamical clusters. Based on this insight, we fixed the values of translational and rotational phoretic mobility parameters to *ζ*_tr_ = 15.4 and *ζ*_rot_ = −0.38, in our simulations and focused on understanding the effect of the interplay between crowding and activity on non-equilibrium structure formation of chemotactic colloids.

Varying Pe and *Φ*, we observe four distinct dynamical steady-states as summarized in the state diagram of Fig. 2. These include active gas, collapsed, dynamical clustering and phase separated states with their representative snapshots shown in the right part of Fig. 2. In a collapsed state, all the particles collapse into a single giant cluster. This state corresponds to the case where *N*^max^_c_/*N* → 1. The hexatic order parameter value in this state is typically *q*_6_ > 0.8. A video corresponding to *Φ* = 0.2 and Pe = 10 in the ESI† shows the collapsed state, where we see fluctuations in the shape of the cluster as well as location and shape of the defects. However, the fluctuations at the interface of the giant cluster are small and only a few particles join and leave the cluster, and the overall number of particles in the big cluster is constant.

In a dynamic clustering state, we observe finite-sized clusters with strong fluctuations in shape and size. We define the dynamical clustering state as a gas of motile clusters with a minimal mean cluster of three and a maximum of 10, *i.e.* 3 ≤ *N*^avg^_c_ ≤ 10. In the phase separated state a big dense cluster coexists with a dilute phase, see the video in the ESI† showing an example for the case *Φ* = 0.2 and Pe = 18. This state is distinguished by a mean cluster size larger than 10. Our choice of this threshold value is somewhat arbitrary, but overall it provides a good distinction between the two states as *N*^max^_c_/*N* is always larger than 0.16 in the phase separated state. The dense cluster resembles to that observed in the motility induced phase separation (MIPS) for purely repulsive active particles. However, here the mechanism of phase separation is fundamentally different as it is driven by the interplay between long-range chemo-phoretic forces ∝ *ζ*_tr_/*r*^2^ and activity. Hence, we refer to it as the chemo-motility induced phase separated (CMIPS) state. We will discuss this state in more detail in section IIIB.

Overall, we observe that upon increasing the Pe, the collapsed state arising from long-range chemo-phoretic interactions melts. However, the observed dynamical state upon an increase of Pe very much depends on the packing fraction. At very low packing fractions *Φ* < 0.01, the increase of the self-propulsion speed beyond Pe > 5 leads to a complete destruction of the structure and appearance of an active gas. For slightly larger packing fractions 0.01 < *Φ* < 0.1, upon increasing the activity, for a small range of Pe, the CMIPS state emerges which transforms into an active gas upon further increase of Pe. For intermediate packing fractions 0.1 ≤ *Φ* ≤ 0.2, upon increasing Pe, the system first undergoes a phase separation at intermediate activities and forms dynamical clusters at higher Pe. At larger packing fractions *Φ* > 0.2, the system undergoes a transition from the collapsed to the CMIPS state and we do not observe any dynamical clustering in the studied range of Pe. The overall emerging trend is that upon increasing the activity, particles can overcome the strongly attractive phoretic interactions, which suppresses the collapsed state and form other dynamical states.

While the collapsed and dynamical clustering states have been reported earlier,^6,23,40^ to the best of our knowledge, this CMIPS state has never been reported before. Therefore, in the subsequent section we will discuss in more detail the mechanisms leading to CMIPS and its distinct characteristics.

### B. Characteristics of the CMIPS state

**Fig. 3:**
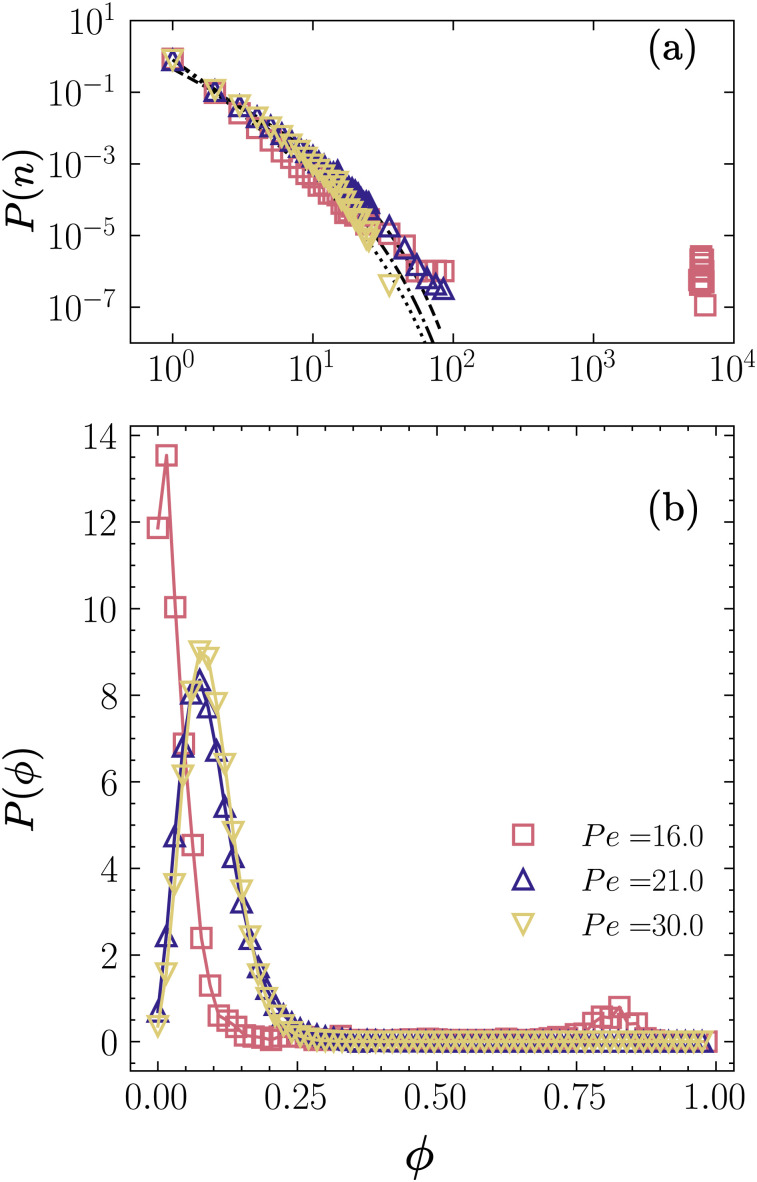
Probability distribution function of (a) cluster-size *P*(*n*) and (b) local packing fraction *P*(*Φ*) for active colloids of overall packing fraction *Φ* = 0.1 at Pe = 16 corresponding to a phase-separated state with mean cluster size *N*^avg^_c_ ≈ 4000, Pe = 21 forming dynamical clusters with *N*^avg^_c_ ≈ 4 and Pe = 30 in a gas state with *N*^avg^_c_ ≈ 2.8. The dashed lines in panel (a) correspond to the fits of *P*(*n*) with the function *a*_0_*n*^−*β*^e^(−*n*/*n*_0_)^ with *a*_0_ = 0.83, *β* = 2.9 and *n*_0_ = 10 for the active gas with Pe = 30, *a*_0_ = 0.5, *β* = 2.01 and *n*_0_ = 10 for the dynamic clusters with Pe = 21 and *a*_0_ = 0.89, *β* = 2.6 and *n*_0_ = 10 for the active gas part of the phase-separated state with Pe = 16.0.

In the CMIPS state, the system phase separates into a large fluctuating cluster which coexists with a dilute fluid. To investigate the statistical features of the CMIPS in Fig. 3 we have presented the extracted distributions of cluster size *P*(*n*) and local packing fraction *P*(*ϕ*) of a CMIPS state occurring at *Φ* = 0.1 and Pe = 16. For comparison, we have also included the corresponding distributions of active gas Pe = 30 and dynamical clustering state at Pe = 21 for the same packing fraction.

In the CMIPS state, *P*(*n*) shown in Fig. 3(a) consists of a broad distribution of small clusters and a single peak corresponding to the phase separated large cluster. In contrast, *P*(*n*) of active gas and dynamical clustering state displays a broad distribution of small clusters which can be described using a power-law exponential curve of the form *a*_0_*n*^−*β*^ exp(−*n*/*n*_0_). The distribution of small clusters for the CMIPS state at *Φ* = 0.1 and Pe = 16 is very similar to that of an active gas demonstrating that the giant cluster coexists with an active gas. Looking into the probability density of the local packing fraction *P*(*ϕ*) shown in Fig. 3(b), we note that the *P*(*ϕ*) of the CMIPS state (*Φ* = 0.1 and Pe = 16) displays two peaks at *ϕ*_1_ ∼ 0.02 < 0.1 and *ϕ*_2_ ∼ 0.85 ≫ 0.1, whereas those of active gas and dynamical clusters exhibit a single peak around *ϕ* ≈ 0.1. The double peak *P*(*ϕ*) confirms the coexistence of a dilute active gas and a dense large cluster for the CMIPS state.

**Fig. 4:**
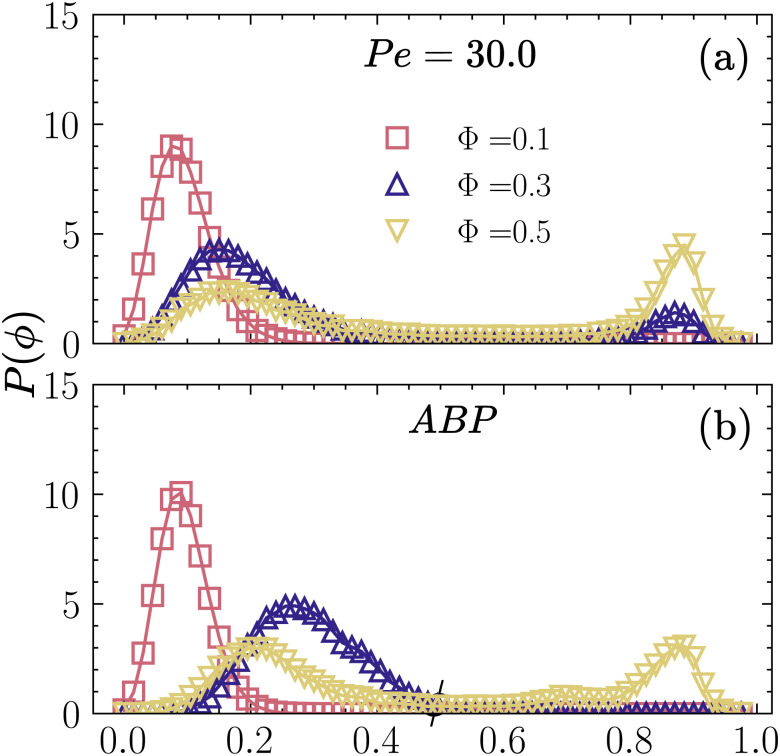
Probability distribution of local density *P*(*ϕ*) at Pe = 30 for (a) self-phoretic colloids at *Φ* = 0.1 (active gas), *Φ* = 0.2 (dynamical clustering) *Φ* = 0.3 and 0.5 (phase-separated states), respectively. (b) Active Brownian particles at *Φ* = 0.1 and 0.3 (active gas) and *Φ* = 0.5 (motility-induced phase-separated state).

The two-peak distribution function for *P*(*ϕ*) is a generic feature for all the CMIPS states observed in our simulations, see Fig. 4(a), for a few examples of the case where Pe = 30. The observed *P*(*ϕ*) for *Φ* = 0.3 and 0.50 is very similar to what is observed for MIPS in purely repulsive ABP systems. For comparison, we have shown the *P*(*ϕ*) of the ABP system for Pe = 30 at *Φ* = 0.1, 0.3 and 0.5 in Fig. 4(b), where only for *Φ* = 0.5, we observe a phase separation, *i.e*, two peaks in *P*(*ϕ*). For self-phoretic active colloids at Pe = 30, the probability distribution function switches from a single-peak to double-peak function for *Φ* > 0.2, where we see a low-density peak around *ϕ*_1_ ∼ 0.15 and a high-density peak around *ϕ*_2_ > 0.8, where the value of *ϕ*_2_ approaches the close packing density *ϕ* ≈ 0.9. For ABPs, the transition of *p*(*ϕ*) from a single-peak to a double-peak occurs at higher densities *Φ* > 0.3, compatible with prior studies.^30^ The curves of *P*(*ϕ*) in Fig. 4 show that in the case of nonequilibrium phase separation, unlike the equilibrium situation, the system does not always separate into the same dilute and dense phases. In particular, the locations of low- and high-density peaks in Fig. 4(a) and (b) depend on the overall packing fraction and are also affected by the nature of interactions.

**Fig. 5:**
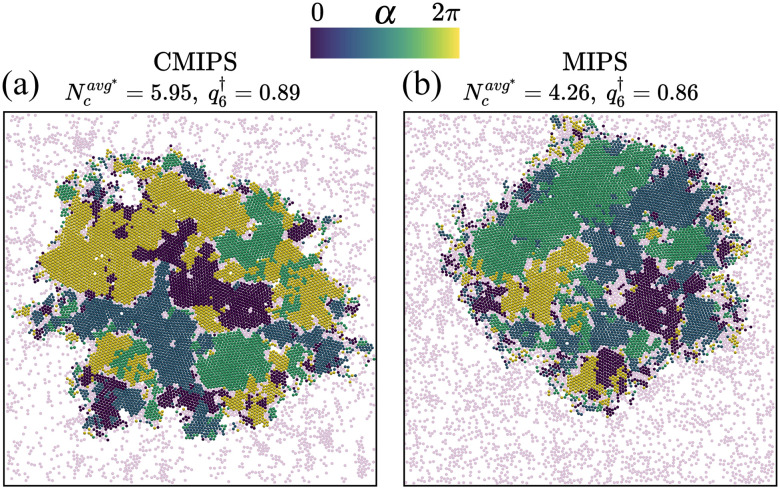
Snapshots of phase-separated states found at packing fraction *Φ* = 0.5 and Pe = 25 for (a) chemotactic and (b) active Brownian particles, where 
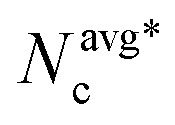
 represents the average cluster size in the dilute phase, and *q*^†^_6_ gives the average hexatic order parameter of the dense giant cluster. The hexatic domains are defined as the regions where |*Ψ*^(*k*)^_6_| > 0.5 and color coded based on the phase *α* of local hexatic order parameter *Ψ*^(*k*)^_6_. Particles colored in light pink are either particles which belong to clusters of 5% or less of the biggest cluster or their hexatic order parameter is |*Ψ*^(*k*)^_6_|<0.5.

At this stage, it is instructive to compare the structural features of the phase separated states for the chemo-phoretic and active Brownian particles at the same *Φ* and Pe. Fig. 5 presents the CMIPS and MIPS states at *Φ* = 0.5 and Pe = 25 where the distribution of hexatic domains according to their orientation have been color coded following the procedure detailed in section IIE. We note that the overall the structure of giant dense clusters in both cases was quite similar. To quantify the degree of order within the dense phase, we compute the magnitude of the hexatic order parameter inside the largest cluster, which we denote by *q*^†^_6_. We obtain similar values *q*^†^_6_ = 0.89 and 0.86 for chemotactic and purely repulsive active colloids, respectively. Likewise, if we compute the mean size of clusters in the dilute phase (outside of the biggest cluster) denoted by 
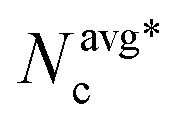
, we obtain 
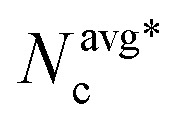
 = 4.95 and 4.26 for the CMIPS and MIPS states, respectively. The attractive phoretic interactions slightly enhance the clustering in the dilute phase, but overall the structures are similar. Because, at such high *Φ*, the steric effects become dominant over the screened phoretic interactions in the dense cluster.

**Fig. 6:**
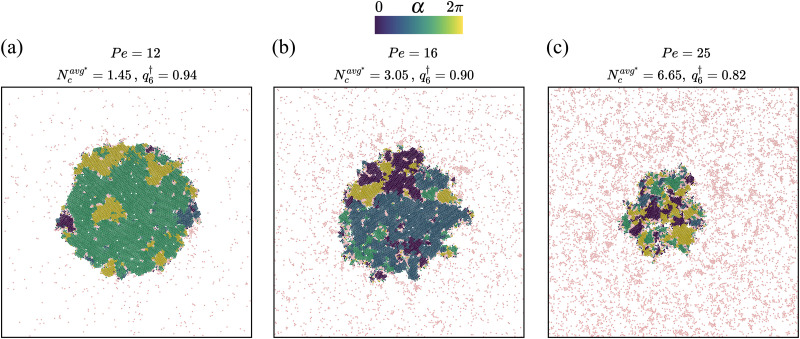
Chemotactic phase-separated states found at packing fraction *Φ* = 0.2 at different activities (a) Pe = 12, (b) Pe = 12 and (c) Pe = 25. Here, 
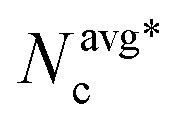
 represents the average cluster size outside the biggest cluster and *q*^†^_6_ gives the mean hexatic order parameter inside the biggest cluster. The colors represent the phase of hexatic domains *α* and light pink colored particles show either particles which belong to clusters of 5% or less of the biggest cluster or particles for which the hexatic order parameter is |*Ψ*^(*k*)^_6_| < 0.5.

The chemo-phoretic interactions however become dominant at lower packing fractions where no MIPS is observed for the ABP system. Fig. 6 displays the the CMIPS states observed at a fixed overall packing fraction *Φ* = 0.2, with varying Pe where the particles are colored according to argument of their *Ψ*_6_. At this *Φ*, we observe a notable change in the structure for both dilute and dense phases upon variation of Pe. At Pe = 12 which is slightly above the largest Pe at which the system collapses, the dense cluster attains a high hexatic order parameter *q*^†^_6_ = 0.94 due to phoretic interactions and the dilute phase is an active gas as evidenced by a low value of 
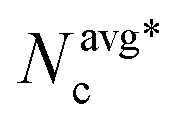
 = 1.45. Upon an increase of Pe, due to competition between chemo-phoretic interactions and activity, the number of hexatic domains increases and *q*^†^_6_ decreases, whereas the mean size of clusters in the dilute phase 
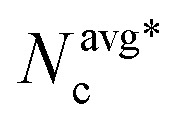
 increases. To summarize, the interplay between long-range phoretic interactions and activity promotes phase separation at relatively low densities and moderate activities whereas at high motilities and low densities, the particles can escape from the dense big cluster; hence a homogeneous gas of dynamical clusters is formed.

## IV. Characteristics of dynamical phase transitions

Having provided an overview of the state diagram, and the salient features of each dynamical state, we focus on understanding the evolution of the relevant order parameters as a function of *Φ* for a wide range of self-propulsion speeds 5 ≤ Pe ≤ 30. We can recognize three distinct regions in the state diagram:

1. At low self-propulsion speeds 5 < Pe < 10, the system goes directly from an active gas to a collapsed state.

2. At intermediate self-propulsion speeds 10 ≤ Pe < 19, the system undergoes a dynamical transition from an active gas to a phase separated state at intermediate packing fractions and finally to a collapsed state at sufficiently high packing fractions *Φ* ≥ 0.50.

3. For Pe ≥ 19, we observe a dynamical transition first from the active gas to dynamical clusters, and then to a phase-separated state up to the highest packing fractions investigated *Φ* = 0.7.

To investigate the nature of dynamical transitions from active gas and dynamical clusters to the phase-separated state, we investigated various structural and dynamical measures as a function of *Φ* at different self-propulsion speeds in the range 10 ≤ Pe ≤ 30.

### A. Mean and maximum cluster size

**Fig. 7:**
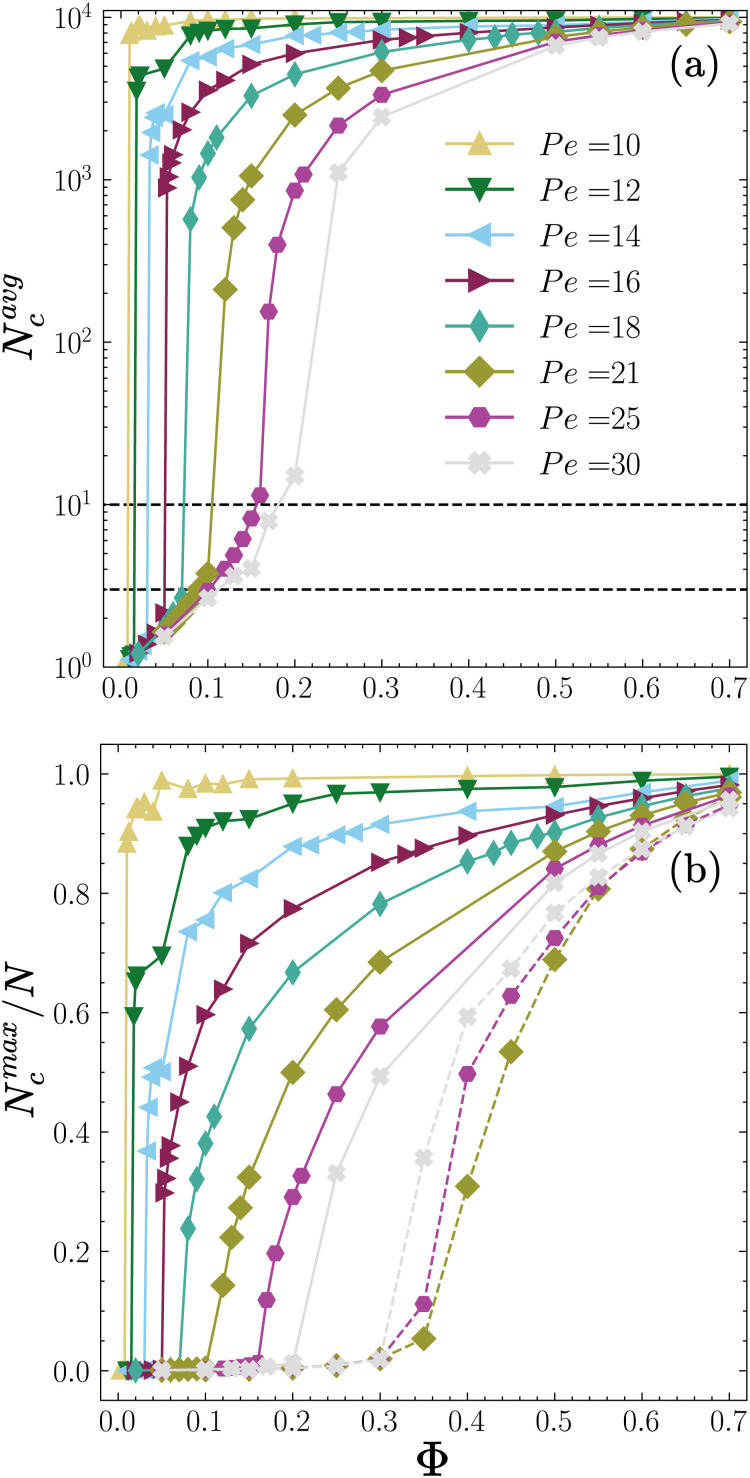
(a) Mean cluster size averaged in time *N*^avg^_c_ and (b) ratio of the largest cluster size averaged in time *N*^max^_c_ relative to the total number of particles *N* = 10^4^ of self-phoretic colloids (*ζ*_tr_ = 15.4 and *ζ*_rot_ = −0.38) as a function of the packing fraction *Φ* for various Péclet numbers 10 ≤ Pe ≤ 30 as given in the legend. The dashed lines in panel (a) correspond to *N*^avg^_c_ = 3 and 10, respectively. The dashed lines in panel (b) show *N*^max^_c_/*N* for ABP particles with periodic boundary conditions with Pe = 21, 25 and 30 with identical color codes as the chemotactic particles.

First, we examine the mean size of the clusters *N*^avg^_c_ and the average fraction of particles in the largest cluster *N*^max^_c_/*N* as shown in Fig. 7(a) and (b), respectively. For Péclet numbers in the range 10 ≤ Pe ≤ 18, we observe a sharp transition of *N*^avg^_c_ from small values *N*^avg^_c_ < 3 to large values *N*^avg^_c_ > 1000 at a low packing fraction *Φ*_L_ < 0.1, the value of which increases with the self-propulsion speed Pe. In particular, at the lowest self-propulsion speed Pe = 10 the sharp transition occurs at a very low packing fraction *Φ* = 0.01. Interestingly, the order parameter *N*^max^_c_/*N* also shows an abrupt jump at the same low *Φ*_L_ < 0.1 followed by a second jump at higher densities *Φ*_H_ > 0.1 for Pe ≤ 16, reminiscent of first-order thermodynamic transitions. The first jump corresponds to a transition from an active gas to a phase-separated state, whereas the second jump demarcates a transition to a collapsed state. For Pe > 20, *N*^avg^_c_ changes more continuously. However, we observe a steep increase in *N*^avg^_c_ around *Φ* = 0.1 where the system goes from an active gas to a dynamic clustering state for which *N*^avg^_c_ > 3. We note that in this regime *N*^max^_c_/*N* remains zero for *Φ* < 0.2 and afterward when the system undergoes phase separation, it continuously evolves akin to a second-order thermodynamic phase transition. Our findings suggest that *N*^max^_c_/*N* is a good order parameter for identification of transition from an active gas or dynamical clustering state to a phase-separated state, although future analysis of the order parameter for different system sizes accompanied by a study of dynamics of transitions is required to establish the existence of a true first- or second-order phase transition.

For comparison, in Fig. 7(b) we have also shown by dashed lines *N*^max^_c_/*N* of the ABP system with periodic boundary conditions at Pe = 21, 25 and 30 where the system undergoes a motility-induced phase separation (MIPS). In agreement with previous reports,^15^*N*^max^_c_/*N* demarcates the transition from a homogeneous fluid to phase separated state when the packing fraction *Φ* is increased. However, we observe a striking difference in the trends. For purely repulsive ABPs, upon increase of activity, the transition occurs at lower packing fractions whereas for chemotactic active particles, the transition shifts to a higher packing fraction at higher Pe. The two opposing trends point to the fundamental difference between mechanisms of phase separation in the two cases. MIPS is promoted by an increase in activity, whereas CMIPS is weakened by an increase in activity as it facilitates escape of particles from the giant cluster formed by chemotactic attractions.

### B. Hexatic order parameter

**Fig. 8:**
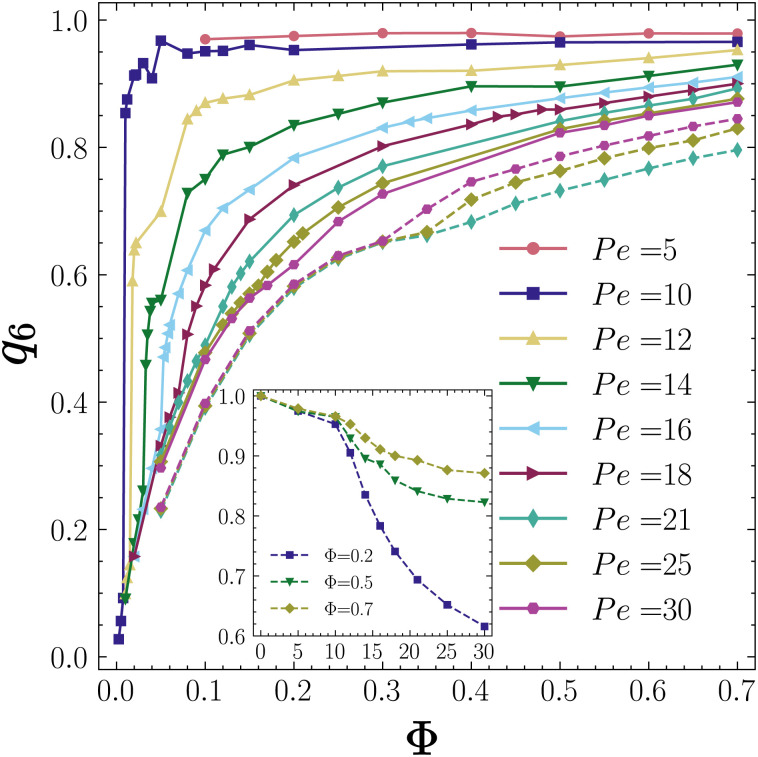
Time-averaged 6-fold-bond orientational order parameter *q*_6_ as a function of the packing fraction *Φ* for Pe = 5, 10, 12, 14, 16, 18, 21, 25 and 30 for chemotactic particles with *ζ*_tr_ = 15.4 and *ζ*_rot_ = −0.38 (continuous lines) and for ABP particles *ζ*_tr_ = 0 and *ζ*_rot_ = 0 at Pe = 21, 25 and 30 (dashed lines). The inset shows *q*_6_ as a function of Pe at *Φ* = 0.2, 0.5 and 0.7.

We have also investigated the global six-fold bond orientation order parameter *q*_6_, see eqn (13), also known as the hexatic order parameter, as a function of the packing fraction at different Péclet numbers. Fig. 8 shows the time-averaged values of the *q*_6_ order parameter as a function of the packing fraction *Φ* at various Péclet numbers for self-phoretic colloids with *ζ*_tr_ = 15.4 and *ζ*_rot_ = −0.38 (continuous lines).

The general trend that we observe is that *q*_6_ increases with *Φ* at each self-propulsion speed. However, at a higher Pe, the value of *q*_6_ at identical packing fractions is lower. We note that for low self-propulsion speeds Pe = 5 and 10 where the system is in a collapsed state already at packing fractions as low as *Φ* = 0.1, *q*_6_ is very close to unity, clear evidence of an overall hexatic order. At this stage, we cannot tell for definite if the system is an active solid or a hexatic liquid as clarifying this requires the calculation of spatial density correlations for very large systems beyond the system size investigated here.^65–67^ However, visual inspections suggest that we have an active solid with long-range positional order.

For 12 ≤ Pe ≤ 16, where the system undergoes a transition from an active gas to a CMIPS state and finally to a collapsed state, we observe a steep increase of *q*_6_ upon phase separation of the system into dense and dilute fluids and a second remarkable increase of *q*_6_ upon entering the collapsed state. The observed trends reinforce the idea that the transitions from an active gas to a phase-separated state and then to a collapsed state are first-order dynamical transitions. At larger self-propulsion speeds, Pe > 18, where the system undergoes a transition from an active gas to a dynamical clustering state, then to a phase separated state, *q*_6_ continuously changes with *Φ* again, consistent with the trends observed for *N*^max^_c_/*N*.

For comparison, we have also included the hexatic order parameter of the ABP system with periodic boundary conditions at Pe = 21, 25 and 30 shown by dashed lines. We note that ABPs generally show a lower degree of hexatic order than chemotactic colloids at identical values of Pe and *Φ*. The value of the hexatic order parameter in all collapsed states is remarkably high, *q*_6_ > 0.85, comparable to the *q*_6_ values obtained in active solids of the ABP system.^67^ This strongly supports our inference that the collapsed state is an active solid. Moreover, if we plot *q*_6_ as a function of Pe at a fixed *Φ* as shown in the inset of Fig. 8 for *Φ* = 0.2, 0.5 and 0.7, we observe a notable reduction in *q*_6_ at the Pe values where the system undergoes phase separation.

### C. Giant fluctuations

One important signature of the nonequilibrium nature of active systems is captured by the giant number fluctuations.^68,69^ In an equilibrium system, the standard deviation Δ*N* of the mean number of particles *N̄* in a subvolume scales as Δ*N* ∼ *N̄*^1/2^ for *N̄* → ∞. However, in an active system this is not true anymore and Δ*N* can scale differently as Δ*N*∼*N̄*^*α*^ with 1/2 < *α* ≤ 1. To examine the effect of chemotactic interactions on density fluctuations, we computed the exponent *α* of giant number fluctuations in subsystems with linear dimensions *L*/*n*, where *n* = 4, 8, 16, 32, and 64. In each case, we evaluated the mean number of particles *N̄* and its standard deviation Δ*N* within each subsystem.

**Fig. 9:**
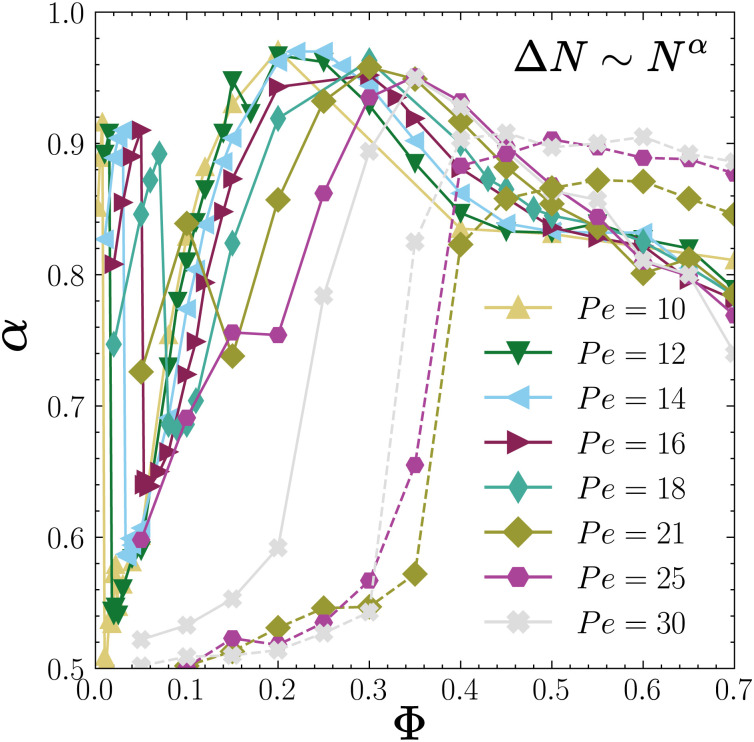
Plot of the exponent *α* of the relation Δ*N* ∼ *N̄*^*α*^ as a function of the packing fraction *Φ* for Pe = 10, 12, 14, 16, 18, 21, 25 and 30 with *ζ*_tr_ = 15.4 and *ζ*_rot_ = −0.38 (continuous lines) and for ABPs with periodic boundary conditions at Pe = 21, 25 and 30 (dashed lines).

Fig. 9 shows the exponent *α* as a function of the packing fraction *Φ* for various Péclet numbers in the range 10 ≤ Pe ≤ 30 for chemotactic colloids with *ζ*_tr_ = 15.4 and *ζ*_rot_ = −0.38 (continuous lines) and for an ABP system in a periodic box (dashed lines). Let us first focus on the region of the phase diagram, 10 ≤ Pe < 18, where the system undergoes two dynamical transitions; first from an active gas to a CMIPS state and then to a collapsed state. For the active gas state, *α* increases systematically with *Φ* from 1/2 to very large values *α* ≈ 0.9 until the system undergoes a phase separation where *α* drops to values below 0.6. Within the phase-separated regime, however, *α* keeps on increasing with *Φ* to very large values *α* ≈ 0.95 until about *Φ* ∼ 0.2 where *α* ≈ 0.95. Afterwards, we observe a decline in *α* until *Φ* ≈ 0.4–0.5, where the system enters a collapsed state. In the collapsed state, it remains nearly constant *α* ≈ 0.4.

For higher Péclet numbers Pe > 18, we observe an increase of *α* with *Φ* as the system goes from an active gas to a dynamical clustering state and then to a CMIPS state up to *Φ* ∼ 0.3. Beyond this point, *α* decreases with *Φ* although it remains significantly larger than 0.5 for all packing fractions. The decrease in *α* beyond *Φ* ∼ 0.3 can be understood in view of increasing size of the largest cluster for large *Φ*. For *Φ* ∼ 0.3 the system is already in a CMIPS state in which a central giant cluster coexists with smaller dynamical clusters and particles continuously join and leave the big cluster. The higher the density, the bigger the central cluster and the smaller the number fluctuations associated with particles joining and leaving the big clusters. This translates into a reduced exponent *α*.

In the case of purely repulsive ABPs, *α* ∼ 0.5 for *Φ* < 0.2 close to its value for the equilibrium case. Upon further increasing the packing fraction, we observe a notable increase of *α* in the region 0.2 < *Φ* ≲ 0.4 concomitant with the occurrence of the motility-induced phase separation. For *Φ* > 0.4, the exponent saturates to a value *α* ∼ 0.9 in agreement with prior results in the literature.^68,70^ Comparing the number fluctuation exponents of chemotactic and active Brownian colloids at high densities reveals that the exponent *α* of ABPs with identical *Φ* and Pe is larger. This indicates that attractive chemotactic interactions reduce the density fluctuations by forming larger clusters as is also visible from larger values of *N*^max^_c_/*N* for the chemotactic system, see Fig. 7(b). A decrease of *α* with *N*^max^_c_/*N* in the phase-separated region is also visible when we consider a fixed *Φ* and increase Pe. Let us consider *Φ* = 0.2 for which we observe a decrease of *α* with increasing Pe. If we compare this trend with the evolution of the phase-separated state with Pe presented in Fig. 6, we note that as we increase Pe, the dilute phase evolves from an active gas to a dynamical clustering state whereas the mean size of the big cluster decreases. In other words, the contrast between the dilute and dense phases decreases with Pe, leading to a decrease in density fluctuations and therefore the exponent *α*.

## V. Concluding remarks

Employing Brownian dynamics simulations, we have explored the non-equilibrium steady-states of a monolayer of self-diffusiophoretic active colloids, in which both translational and rotational degrees of freedom of particles are coupled to a chemical field. Assuming that the chemical field diffuses much faster than the colloids, we adopted a stationary profile of the chemical field.^23,39^ We focused on the case when chemical-mediated interactions induce effectively attractive forces and repulsive torques leading to the formation of dynamical clusters at low densities and sufficiently large activities as confirmed from experiments^7,43^ and simulations.^23,39^ We investigated the collective organization of self-diffusio-phoretic colloids at intermediate and high packing fractions which had not been investigated before. In this work, we present a comprehensive state diagram as a function of the packing fraction and the Péclet number (Pe) while keeping the chemotactic parameters fixed. We find that the interplay between steric, chemo-phoretic interactions and activity, gives rise to the emergence of a new phase-separated state which we coined the chemo-motility induced phase-separated (CMIPS) state. This state is observed for a wide range of packing fractions and it can appear at surprisingly low packing fractions and moderate Péclet numbers. Similar to the MIPS observed for active particles with purely excluded volume interactions^30^ and with short-ranged attractive interactions,^37^ the CMIPS state consists of a dense fluid coexisting with a dilute phase. As revealed by our quantitative analysis, presented in Fig. 5, the dense cluster of CMIPS is structurally similar to that of MIPS. However, underlying mechanism of phase separation for chemotactic active colloids is different.

MIPS is governed by a self-trapping mechanism where collision of rapidly propelled but slowly reorienting particles leads to slowing down and clustering. The colliding particles entrapped in a cluster provide a seed point for other particles joining the cluster to get ‘stuck’. This process creates a positive feed-back loop through which more particles accumulate in denser regions and thus, slow down, eventually triggering a complete phase separation between a slow-moving dense fluid and a fast-moving dilute phase.^8–10^ CMIPS, on the other hand, is triggered by the interplay between long-range attractive phoretic interactions causing condensation of the system (collapse) and activity which results in evaporation of particles from the collapsed dense cluster. This different mechanism leads to the formation of CMIPS at packing fractions and activities notably lower than those for which MIPS is observed.^11,30,37,67^ Remarkably, the CMIPS state can occur at packing fractions as low as *Φ* ≈ 0.01 for intermediate Péclet numbers and calls for new experiments of chemotactic active colloids to detect this state.

Our study also shows that the dynamical transition from an active gas or dynamical clustering state to the CMIPS state upon increasing the packing fraction can be determined from the mean fraction of particles in the largest cluster *N*^max^_c_/*N*. At intermediate Pe where the system undergoes a transition from an active gas to CMIPS state, *N*^max^_c_/*N* displays an abrupt jump whereas at higher Pe where the system enters a phase-separated state from a dynamical clustering state, *N*^max^_c_/*N* changes continuously with *Φ*. The observed trends are reminiscent of a first- and second-order thermodynamic phase transitions but a more systematic study of transition kinetics and dependence of order parameter on system size is required to establish the true nature of dynamic phase transitions. We also observe a qualitative difference with MIPS in dependence of *N*^max^_c_/*N* on activity. Upon increasing the activity at the same packing fraction, CMIPS is weakened and the dense cluster becomes progressively smaller and less ordered, whereas MIPS is promoted by an increase in activity leading to a larger *N*^max^_c_/*N* and a larger degree of hexatic order in the dense phase.

At this point, it is worthwhile to discuss similarities and differences between the collective organization of chemo-phoretic colloids interacting *via* long-range isotropic attractive forces and weak repulsive torques and active colloids interacting *via* short-range attractive interactions.^22,36–38^ In semidilute active colloids, *Φ* ∼ 0.1, with isotropic Lennard-Jones interactions, the competition between attraction and self-propulsion leads to cluster formation.^36,38^ Similar to low-density chemotactic colloids, comparable attraction and propulsion strengths lead to formation of steady dynamical clusters, whereas predominance of attractive over self-propulsive forces promotes clusters which coarsen over time at a rate depending on the strength of attraction. However, even at the largest activities and longest times, the mean cluster size of coarsening clusters is about 100 for a system of *N* = 10 000 particles and a rapid macroscopic phase separation similar to CMIPS is not observed. We note that anisotropy of chemotactic interactions in our study enters only *via* weak repulsive torque in contrast to active amphiphilic patchy particles^38^ and active dipolar colloids,^28^ where the directionality of interactions is manifested in both attractive forces and torque. In the latter case, orientation-dependent interactions strongly affect the morphology of aggregates in a semi-dilute regime, *e.g.*, leading to the formation of string-like aggregates, whereas morphology of chemotactic clusters is isotropic.

Now, if we consider moderate packing fractions, we notice remarkable differences in the collective organization between active colloids with short-range attractions^37^ and those with long-range chemotactic attractions. At *Φ* = 0.4, active colloids with strong short-range attractions^37^ undergo a transition from a system-spanning ramified network structure (microphase separation) at low activities to a homogeneous state at intermediate activities and finally at high activities exhibit a reentrant phase separation, however, now a macroscopic one where a dense fluid coexists with a dilute gas. On the other hand, chemotactic active colloids interacting with long-range attractive forces, aggregate at low activities into a giant solid-like cluster (collapse) which phase separates into a dense and dilute fluid at moderate and high activities. Although in both cases, the competition between self-propulsive and attractive forces affects the structure formation, the sequences of observed phases are very different.

To conclude, our results for a stationary chemical field highlight the role of long-range chemical field-mediated interactions in inducing phase separation of self-phoretic active colloids at relatively low self-propulsion speeds and remarkably low packing fractions. Statistical features of CMIPS pose open questions which merit further investigation in the future to shed more light on similarities and differences between CMIPS and MIPS. These include establishing a link between spontaneous velocity alignment in the dense cluster and phase separation^32^ and the universality class of this dynamical phase transition.^31,34^ In a study where the full time-dependent solution of the chemical field coupled to the orientational degrees of phoretic colloids was considered,^41^ interesting wave patterns resulting from delay effects emerged. It remains open as to what dynamical patterns in denser systems emerge when both translational and rotational degrees of freedom are coupled to non-stationary chemical field gradients. In the future, we plan to extend this work to consider an explicit solution of the dynamical equation of the chemical field, where similar to references 41 and 71 screening of the chemical field is directly implemented.

## Data availability

The data that support the findings of this study are available from the corresponding author upon reasonable request.

## Conflicts of interest

There are no conflicts to declare.

## Supplementary Material

SM-019-D2SM00957A-s001

SM-019-D2SM00957A-s002
